# An integrative imputation method based on multi-omics datasets

**DOI:** 10.1186/s12859-016-1122-6

**Published:** 2016-06-21

**Authors:** Dongdong Lin, Jigang Zhang, Jingyao Li, Chao Xu, Hong-Wen Deng, Yu-Ping Wang

**Affiliations:** Department of Biomedical Engineering, Tulane University, New Orleans, LA 70118 USA; Center for Bioinformatics and Genomics, Tulane University, New Orleans, LA 70112 USA; Department of Biostatistics and Bioinformatics, Tulane University, New Orleans, LA 70112 USA

**Keywords:** Multi-omics data, Imputation, Integrative analysis, Ensemble learning

## Abstract

**Background:**

Integrative analysis of multi-omics data is becoming increasingly important to unravel functional mechanisms of complex diseases. However, the currently available multi-omics datasets inevitably suffer from missing values due to technical limitations and various constrains in experiments. These missing values severely hinder integrative analysis of multi-omics data. Current imputation methods mainly focus on using single omics data while ignoring biological interconnections and information imbedded in multi-omics data sets.

**Results:**

In this study, a novel multi-omics imputation method was proposed to integrate multiple correlated omics datasets for improving the imputation accuracy. Our method was designed to: 1) combine the estimates of missing value from individual omics data itself as well as from other omics, and 2) simultaneously impute multiple missing omics datasets by an iterative algorithm. We compared our method with five imputation methods using single omics data at different noise levels, sample sizes and data missing rates. The results demonstrated the advantage and efficiency of our method, consistently in terms of the imputation error and the recovery of mRNA-miRNA network structure.

**Conclusions:**

We concluded that our proposed imputation method can utilize more biological information to minimize the imputation error and thus can improve the performance of downstream analysis such as genetic regulatory network construction.

**Electronic supplementary material:**

The online version of this article (doi:10.1186/s12859-016-1122-6) contains supplementary material, which is available to authorized users.

## Background

Recent advances in high-throughput technologies prompt the production of a variety of ‘omics’ data such as transcriptomics, proteomics and metabolomics from the same set of subject tissues/cells, facilitating the discovery of various levels of risk genetic factors for the analysis of human complex diseases. However, due to technical limitations of these high throughput technologies and experimental designs, the presence of missing values remains an inevitable and prevalent problem in large-scale profiling experiments [1]. For example, proteomics data suffers significantly from missing values due to the imperfect identification of coding sequences within a genome and the limited sensitivity of current peptide detection technologies [2–4]. Current technologies allow the detection of only one-third to one-half of all coded proteins and thus leave a significant number of proteins experimentally undetected [5–7]. In miRNA array, it is often observed that a large portion of miRNAs are expressed below the detection limit, resulting in missing data in the output [8, 9]. In general, there are three types of missing mechanisms [10, 11]: the first one is data missing completely at random (MCAR), where data missing is due to some factors unrelated to the experimental questions. The causes of missing are usually unobserved in the experiment. The second mechanism is data missing at random (MAR), where missing depends on some variables, which can be measured in the experiment such as different slides, media or experimental conditions for assaying expression data. The last one is missing not at random (MNAR) where data missing is caused by some unobserved variables; they may be highly related to the experiment, for instance, low-abundance expression may remain undetected due to the detection bias of instruments.

A number of studies have indicated that missing values in large-scale omics data can drastically hinder downstream analyses, such as unsupervised clustering of genes [12], detection of differentially expressed genes [13], supervised classification of clinical samples [14], construction of gene regulatory networks [15], genome wide association studies [16] and detection of differentially methylated regions [17]. Missing values in multi-omics data can also obstruct integrative analysis of multi-omics data, leading to difficulty in the interpretation of complex diseases. Therefore, it is highly demanded to impute the missing values before performing integrative analysis of multi-omics data.

However, most current imputation methods mainly focus on single omics data as reviewed in [18], including global methods such as Bayesian principle component analysis (BPCA) [19], and singular value decomposition imputation (SVDimpute) [20], local methods such as k nearest neighbor imputation (KNNimpute) [20], local least square imputation (LLS) [21] and iterative local least square imputation (iLLS) [22], and hybrid methods which combine both global and local methods such Lincmb [23]. The main limitation of these imputation methods is that they only focused on utilizing the information from single omics data. Thus there is an increasing interest in incorporating additional information for the imputation, e.g., biological databases or other omics data [24]. For transcriptomics datasets, a priori information about the functional similarities in term of GO (Gene Ontology) was used for missing value imputation, based on the idea that functionally related genes tend to express in a modular fashion [25]. Experimental results indicated that the imputation accuracy can be enhanced by incorporating GO information, even when the missing rate was large [26, 27]. Other knowledge based impute methods such as integrative missing value estimation method (iMISS) employed the information from multiple external references data to find consistent and reliable neighboring genes of a target missing gene for better estimation [28]. Meta-data imputation method combined multiple available microarray datasets of a species to select top closest columns to impute missing column in target missing matrix [29]. These knowledge-based imputation methods usually require the features (e.g., genes) correspond with each other across diverse datasets and they still focus on one type of omics data. There are some endeavors to incorporate the relationships between diverse omics data into the imputation. Nie et al. proposed a Zero-inflated Poisson regression model to use the correlation between transcriptomics and proteomics datasets for imputing the missing proteomics data [30]. Torres-Garcia et al. published a stochastic Gradient Boosted Trees (GBT) approach to uncover possible nonlinear relationships between transcriptomics and proteomics data. GBT was used to predict those protein abundance not experimentally detected based on the predictors such as mRNA abundance, cellular role, molecular weight, sequence length, protein length, GC content and triplet codon counts [4, 31]. Histone acetylation information was combined into KNNimpute and LLS to improve the missing value estimation of gene expression data [32]. Artificial neural network approach was also applied to impute the missing values of the proteins using the relations between transcriptomics and proteomics data in the study [33].

By extending these methods to incorporate informative features from other types of omics data for the imputation, we developed an ensemble learning based algorithm to deal with missing values. Our multi-omics imputation method takes advantage of the correlation across different omics data with the assumption that the missing feature from one type of omics data can be explained by its neighboring features from the same omics data as well as the features from other omics data. Some prior biological knowledge about interactions among different levels of omics data (e.g., GO, protein-protein interaction database) can also be incorporated. In addition, to account for the situation that there are multiple omics data having missing values, we extended our multi-omics imputation method to simultaneously impute multiple missing omics data. We compared our method with five single omics data imputation methods with respect to different noise levels, sample sizes and missing rates. Moreover, we further evaluated the impact of different imputation methods on downstream analysis, e.g., mRNA-miRNA network reconstruction. The results consistently confirmed the advantage and efficiency of our multi-omics imputation method in terms of the imputation error and the recovery of mRNA-miRNA network.

## Methods

In this study, we take three kinds of omics data (e.g., mRNA, microRNA and DNA methylation) as an example to elaborate our method. For each omics data, it is represented by a matrix ${G}_i\in {R}^{p_i\times n},i=1,2,..,m$, where *i* indicates the type of omics data, *p*_*i*_ is the number of rows of each matrix corresponding to different types of features (e.g., gene expression) and *n* is the number of columns corresponding to different subjects. The missing point at the *m*-th feature on the *l*-th subject is denoted by *G*_*i*_^*m*,*l*^, m = *1*, *2*, …, *p*_*i*_, *l* = *1*, *2*, …, *n*. In the following, we first introduce diverse single omics imputation methods, and then propose multi-omics data imputation and its extension to more general case.

### Single omics imputation

For each single omics data matrix, global methods (e.g., BPCA and SVDimpute) and local methods (e.g., KNNimpute, LLS and iLLS) were developed to explore neighboring global or local features to impute missing features, please refer to Additional file 1 A for details. Without the loss of generality, we assume that the target gene *g*_*t*_ ∈ *R*^*n*^ in ${G}_1\in {R}^{p_1\times n}$ contains missing values located in the first *s* subjects. Hence,

1 $$ {\mathrm{g}}_{\mathrm{t}}=\left[{\mathrm{g}}_{\mathrm{t}}^{\mathrm{miss}},{\mathrm{g}}_{\mathrm{t}}^{\mathrm{c}}\right] $$

where *g*_*t*_^*miss*^ ∈ *R*^1 × *s*^ is the missing vector in the target gene and *g*_*t*_^*c*^ ∈ *R*^1 × (*n* − *s*)^ is complete vector containing non-missing values. To estimate the missing vector *g*_*t*_^*miss*^, firstly, we compute the distance (Euclidean distance) *d*_*t, j*_ between the target gene *t* and other gene *j* (or eigengene *j* [20]); secondly, top *k* close genes (or eigengenes), denoted by *Gk* = [*Gk*^*miss*^, *Gk*^*c*^] ∈ *R*^*k* × *n*^ are used for imputation. Specifically, KNNimpute estimates g_t_^miss^ by averaging the weighted values of neighboring genes or eigengenes while the other methods tend to use linear regression as in (2)

2 $$ {\overset{\sim }{g}}_t^{miss}=\frac{{\displaystyle {\sum}_{j=1}^k}G{k}_j^{miss}/{d}_{t,j}}{{\displaystyle {\sum}_{j=1}^k}1/{d}_{t,j}}\kern0.28em \mathrm{or}\kern0.28em {\overset{\sim }{g}}_t^{miss}=G{k}^{miss}\times \beta $$

where *Gk*^*miss*^ ∈ *R*^*k* × *s*^ is the submatrix of *Gk* corresponding to the missing location in the target gene; and *β* is the coefficient vector to weight the contribution of neighboring genes/eigengenes, which can be estimated by the following least square minimization:

3 $$ argmi{n}_{\beta }{\left\Vert {g}_t^c-G{k}^c\times \beta \right\Vert}_2^2 $$

Therefore, the missing values can be approximated by *β* = (*Gk*^*c*^)^†^*g*_*t*_^*c*^, where (*Gk*^*c*^)^†^ is the pseudo inverse of *Gk*^*c*^.

### Multi-omics data imputation

**Fig. 1:**
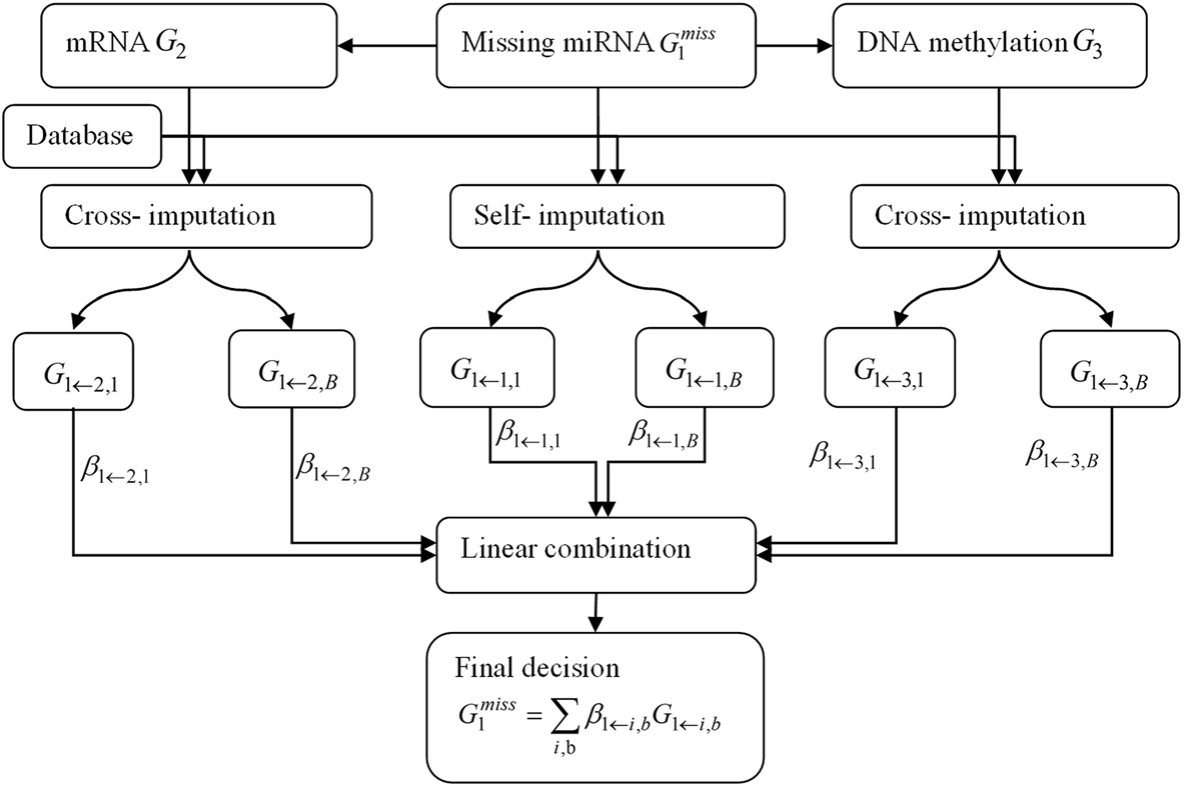
Schematic representation of multi-omics imputation method

Instead of imputing each omics data separately, we proposed to combine multiple information from various omics data such as microRNA (*G*_1_), mRNA (*G*_2_) and DNA methylation (*G*_3_), which have been identified to be correlated with each other in their elements or components [34, 35]. As shown in Fig. 1, we built an integrative model based on ensemble learning [36, 37], which generally consisted of three steps: the first step was ensemble learning which generated a set of basic models; the second was the ensemble pruning, where models were pruned to remove some models with little contributions; the final step was the integration of multiple models into a new prediction model. In this study, we built a predictive model on a set of basic models for missing value estimation, as shown in Fig. 1. Multiple constraints were imposed on each basic model (e.g., non-negativity constraint) to reduce the overfitting as well as the influence of those basic models with little contribution.

The basic models were generated based on three types of imputations, i.e., self-imputation and cross imputation by *G*_2_ and *G*_3_ respectively. The self-imputation was to impute *G*_1_ by itself using single-omics imputation method as mentioned in the section of “Single omics imputation”. The cross-imputation was to impute *G*_1_ by other omics data, i.e., *G*_2_. Because of the scale difference among different types of omics data, we intended to impute each missing feature in G_1_ individually by exploiting the correlated information from *G*_2_. For each target gene *g*_*t*_ = [*g*_*t*_^*miss*^, *g*_*t*_^*c*^] in *G*_1_, it was combined with correlated features in *G*_2_ to be a new missing matrix *H*. Matrix *H* was then imputed by self-imputation methods to estimate *g*_*t*_^*miss*^. Eventually, we obtained three imputation outputs for all missing values in *G*_1_ by different omics data, denoted by *G*_1 ← 1_, *G*_1 ← 2_ and *G*_1 ← 3_ respectively. Moreover, prior knowledge from accessible databases can also provide extra information (e.g. protein-protein interactions (PPI), co-expressed genes) to improve the imputation accuracy. In this study, we took advantage of information from PPI to partially avoid the overfitting for LLS and iLLS in *G*_2_. For example, to impute the expression data of target gene *g*_*t*_ by other genes in *G*_2_, we collected those genes that had STRING scores > 0.9 [38, 39] with *g*_*t*_ in STRING database and had significant correlation (*p* < 0.05) in *G*_2_. We keep these genes in the prediction model for LLS and iLLS during the feature selection procedure. This partially decreased the selection of genes which had random correlations with target gene in *G*_2_. To further improve the accuracy of prediction, we considered the generation of heterogeneous learning algorithms that can ensure a level of diversity among the basic models. Diverse models can provide different predictions, which can be combined for better performance. We subsampled features in omics data and re-run the imputation *B* times to get multiple imputed matrices, {*G*_1 ← 1,*b*_, *G*_1 ← 2,*b*_, *G*_1 ← 3,*b*_}, *b* = 1, 2, …, *B*.

To integrate multiple imputation models, we used a least square regression model to combine the outputs from diverse models as

4 $$ \begin{array}{l} \min {{\displaystyle \sum_j\left[{G}_1^j-{\displaystyle \sum_{i,b}{\beta}_{1\leftarrow i,b}{G}_{1\leftarrow 1,b}^j}\right]}}^2\\ {}s.t.\kern0.5em {\displaystyle \sum_{i,b}{\beta}_{1\leftarrow i,b}}=1,\kern1em {\beta}_{1\leftarrow i,b}\ge 0,i=1,2,\dots, m,b=1,2,\dots, B\end{array} $$

where *β*_1 ← *i*,*b*_, *i* = 1, 2, …, *m*, *b* = 1, 2, …, *B* are the weights for different basic imputation models, and *j* indicates missing location in target gene. Since all these models aim to impute the same missing values, their outputs are highly correlated. Instead of using ridge regression, we imposed non-negative regularization on the coefficients to handle the high multi-colinearity among variables in the model, which has been found to be more reliable and consistent [37]. To avoid the over-fitting issue, we adopted bootstrapping to randomly generate faking missing values at the locations which were not overlapped with true missing locations. The weights were estimated by (4) based on the imputed and true values on the faking missing points (Additional file 1 B). The averaged value of each weight on *T* times bootstrapping was used for prediction. We set *T* to be 30 in the following experiments.

### Extension of multi-omics data imputation

For integrative analysis of multi-omics data, there are usually missing values on each individual omics data. To handle this situation, we extended our multi-omics imputation method by incorporating an iterative method to simultaneously impute each omics data. The iterative procedure is shown in Table 1. There are two parts in our iterative multi-omics imputation algorithm. The first one is updating each omics data sequentially within the iteration and second one is an iterative procedure. Within each iteration, we impute each missing omics data separately but following a specific order of the number of missing genes from smallest to the largest (i.e. miRNA to mRNA), similar to sequential KNN [40] or sequential LLS impute [41] methods. This is expected to control the propagation of imputation errors from smallest to largest. After one omics data is imputed, the new completed matrix can be used for other omics data imputation to reduce the error. When all of omics data are imputed once, they can be reused to refine the prediction of missing values, as suggested in iterative LLS, iterative KNN [42] and iterative biclustering imputation methods [43].

**Table 1:** Algorithm for iterative multi-omics imputation

A: Initialize with replacing all missing values in all matrices *G*_*i*_, *i* = 1, 2, 3 by self-imputation methods to obtain complete matrices {*G*_*i*_^(0)^}.
B: for each iteration h,
(1).
a. Self-impute *G*_1_ based on *G*_1_^(*h* − 1)^; Cross-impute *G*_1_ by *G*_2_^(*h* − 1)^, *G*_3_^*h* − 1^ using multi-omics imputation method to obtain *G*_1_^(*h*)^.
b. Self-impute *G*_2_ based on *G*_2_^(*h* − 1)^; Cross-impute *G*_2_ by *G*_1_^(*h*)^, using multi-omics imputation method to obtain *G*_2_^(*h*)^.
c. Self-impute *G*_3_ based on *G*_3_^(*h* − 1)^; Cross-impute *G*_3_ by *G*_1_^(*h*)^, *G*_2_^(*h*)^ using multi-omics imputation method to obtain *G*_3_^(*h*)^.
(2). Determine the sum of square of difference on the missing locations j between {*G*_*i*_^(*h* − 1)^} and {*G*_*i*_^(*h*)^}:
$$ {\delta}^h={\displaystyle \sum_j{\displaystyle \sum_i{\left({G}_i^{j,\left(h-1\right)}-{G}_i^{j,(h)}\right)}^2}} $$
C. If *δ* ^*h*^ ≤ *τ*, the iteration is stopped and output {*G*_*i*_^(*h*)^}; otherwise go to Step 2 to continue the iteration until the convergence criteria *τ* is reached.

In the simple case that only one omics data contains missing values, there is only one step in the iteration. In the case of missing values in multiple omics data, compared with performing single omics imputation separately, the advantage of our extended multi-omics imputation is its ability of reusing data in both self-imputation and cross-imputation processes. In current single-omics imputation methods, most of them use row average to impute missing value initially for deriving the neighboring gene/eigengene only once, which can cause biases in the final imputation. Instead of using one-time row average initialization, in each iteration, the self-imputation (e.g., *G*_1 ← 2_^(*h*)^) is implemented based on the completed matrix from the previous iteration *G*_1 ← 2_^(*h* − 1)^, which is updated iteratively to reduce the bias. In addition, information from other omics data will be incorporated by the cross-imputation, which can further improve the imputation accuracy, especially when large missing data exist in *G*_1 ← 2_^(*h*)^.

### Evaluation metric

We compared the performance of our proposed multi-omics-based imputation with single-omics-based imputation methods. The performance was evaluated by the normalized root mean squared error (NRMSE) as follows:

5 $$ NRMSE=\sqrt{\frac{\frac{1}{N}{\displaystyle \sum_i{\displaystyle \sum_j\left({G}_i^j-{\widehat{G}}_i^j\right)}}}{\operatorname{var}\left({G}_i^j\right)}} $$

where *G*_*i*_^*j*^ and *Ĝ*_*i*_^*j*^ are the true and imputed value of the *j*-th missing point in the *i*-th omics data respectively; and N is the number of missing points in all datasets. In addition, we performed a paired *t*-test for each factor exploration, measuring the significance of NRMSE difference between the methods along with each simulation.

## Results

### Simulation scheme

We performed simulation analysis to evaluate the performance of our proposed methods based on the MCAR missing mechanism. Simulation data were derived from the cancer genomic atlas (TCGA; http://cancergenome.nih.gov/) database on Glioma cancer study containing 50 subjects with 5939 mRNAs, 104 microRNAs and 5013 DNA methylation sites. We have removed missing values in all of these data, yielding complete data matrices. Based on these observation matrices, a certain percent of entries (e.g., 1, 5 %) was randomly set to have missing values. To evaluate the effect of sample size (e.g., 10, 20 subjects), a specific number of samples was first selected randomly and then missing matrices were generated. Similarly, for different noise levels, a random noise from normal distribution *N*(0, *σ*_*e*_^2^) with different standard deviations was added to the observed matrices for missing matrices generation. Each type of missing matrix generation was repeated 50 times, and then several imputation methods were applied for comparison.

First, we compared the single-omics based method with multi-omics based imputation method when only one type of omics data (e.g., miRNA) contained missing values and the other datasets (e.g., mRNA and DNA methylation) were complete. Then, to consider more realistic situation when more than one type of omics data contained missing values, we simulated both mRNA and miRNA datasets with missing values. For simplicity, we set the same missing rate on both datasets. Single-omics-based method was applied to each type of missing matrix, while our iterative multi-omics method was directly used for imputing both datasets simultaneously. Finally, the imputation accuracy was evaluated on both simulations.

### Parameters setting

For KNNimpute method, we set the neighboring size to be 15 as suggested in [20, 28]. Both BPCA and SVDimpute depend on the number of principal axes (eigenvectors). The number of eigenvectors selected in SVD imputation was set to be 20 % of the number of samples as tested in [20]; but the number of PCs used in BPCA could be set more loosely as suggested in the earlier study [19] which suggested a safety number of PCs to be *k* = D-1,where D was the number of samples. The automatic relevance determination prior applied in Bayesian estimation can reduce the redundant dimension automatically. For local regression based methods, LLS impute [21] and iLLS impute [22], a procedure of estimating the optimal number k was applied. Prior to imputation, missing values were initially estimated by row average and some faking missing values were generated with true values known. The methods searched optimal *k* (LLS) or ratio (iLLS) value from a given range (e.g., *k* between 2 and D-1 or ratio between 0 and 1) with the lowest estimation error.

### Comparison on single missing omics data

We simulated one omics data (e.g., miRNA) with missing values while keeping the other omics datasets (e.g., mRNA and DNA methylation) to be complete. Five popular imputation methods were applied to single missing omics data, which were then compared with our proposed multi-omics imputation method. Three simulations were performed to study the effects of three factors on imputation accuracy: missing rate, sample size and noise level.

### Effect of missing rate

**Fig. 2:**
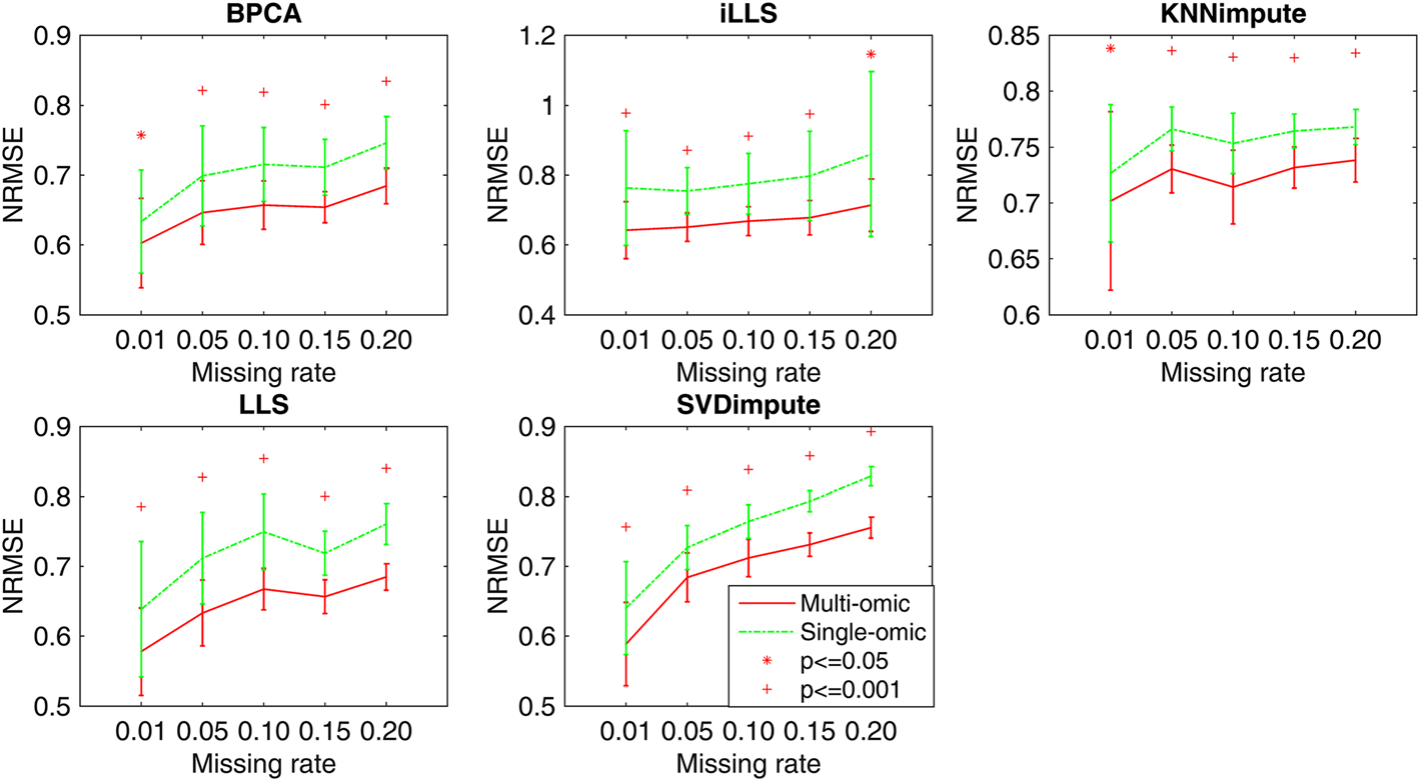
Average NRMSE by five imputation algorithms (BPCA, iLLS, KNNimpute, LLS and SVDimpute) on single omics data *v.s.* multiple omics datasets with different missing rates (e.g., 1, 5, 10, 15 and 20 %)

We randomly generated missing values in miRNA matrix with different percent of missing rate (e.g., 1, 5, 10, 15 and 20 %) for comparison. Figure 2 shows the performance of five imputation methods applied to single missing omics data and compared to our method using multiple omics data with different missing rates. The results show significant decrease of NRMSE with our multi-omics based method over five prevalent methods for single-omics. This improvement is demonstrated consistently across all different missing rates, showing the efficiency of our method in utilizing complementary information from mRNA and DNA methylation dataset. With the increase of missing rate, the performance of all methods is degraded with increasing NRMSE. Especially for global algorithms, i.e., BPCA and SVDimpute, the decrease of NRMSE is more significant than using local algorithms, indicating the larger effect of missing data rate on using global features (e.g., principle components of data matrix) than that on local feature selection. However, this decrease is reduced by using extra information from other datasets in our multi-omics imputation, indicating the advantage of using multi-omics data in our method over single-omics method.

#### Effect of sample size

**Fig. 3:**
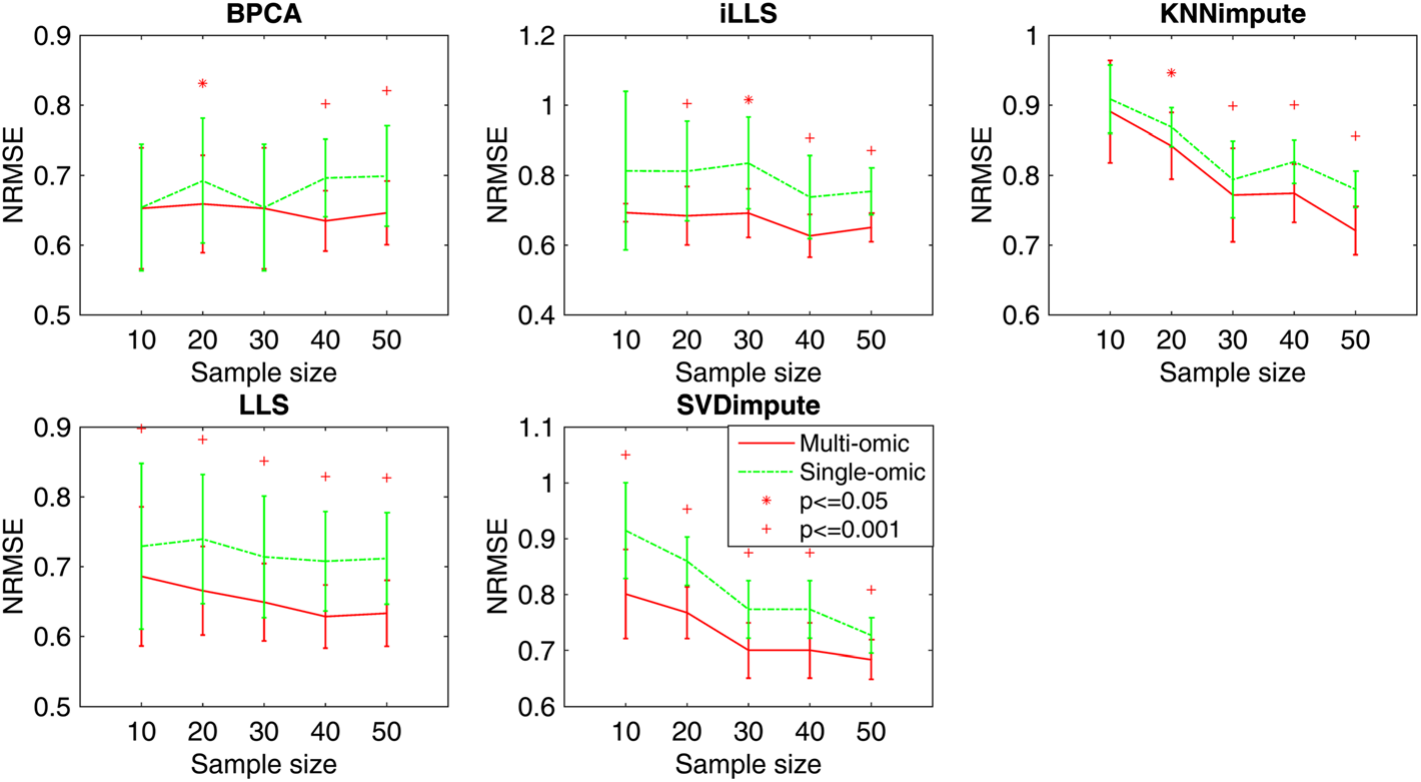
Average NRMSE by five imputation algorithms (BPCA, iLLS, KNNimpute, LLS and SVDimpute) on single omics data v.s. multiple omics datasets with different sample size (10, 20, 30, 40 and 50)

Imputation accuracy may be influenced by the number of samples in the missing data. Too few samples can lead to large variances of the imputed values. For local algorithms, mostly based on regression models, few sample size will lead to the increases of the variance of estimated coefficients; for global algorithms, the matrix tends to be ill-conditioned if the sample size is limited. To evaluate the effect of sample size on the performance of our imputation method, we simulated five datasets with sample size from 10 to 50, and randomly set 5 % missing values in each dataset. Figure 3 shows the influence of sample size on both single-omics and multi-omics imputation methods. It can be seen that our multi-omics method consistently outperforms single-omics method with respect to all sample sizes, indicating the benefit of incorporating extra information, especially when the sample size is small. In most algorithms, both the imputation error and variance are generally decreasing with the increase of sample size, showing the importance of sample size for reducing imputation errors. For iLLS and LLS, the gain of multi-omics method over single omics method is relatively stable regardless of sample size. However, for KNNimpute and SVDimpute algorithms, the influence of sample size is significant, in that NRMSE decreases rapidly by increasing sample size. In particular, when sample size is small (<30), the NRMSE of single-omics method increases significantly while our multi-omics method shows less increase, demonstrating the advantage of multi-omics method.

#### Effect of noise level

Due to technical limitations, there are a variety of noises introduced in the collected data, which may cause difficulty in imputation. To test the robustness of diverse imputation methods to noise, we simulated five datasets by adding different levels of Gaussian noise with varying standard deviations (std) from 0.1 to 1. The sample size was set to be 50 and missing rate was 5 % in all datasets.

**Fig. 4:**
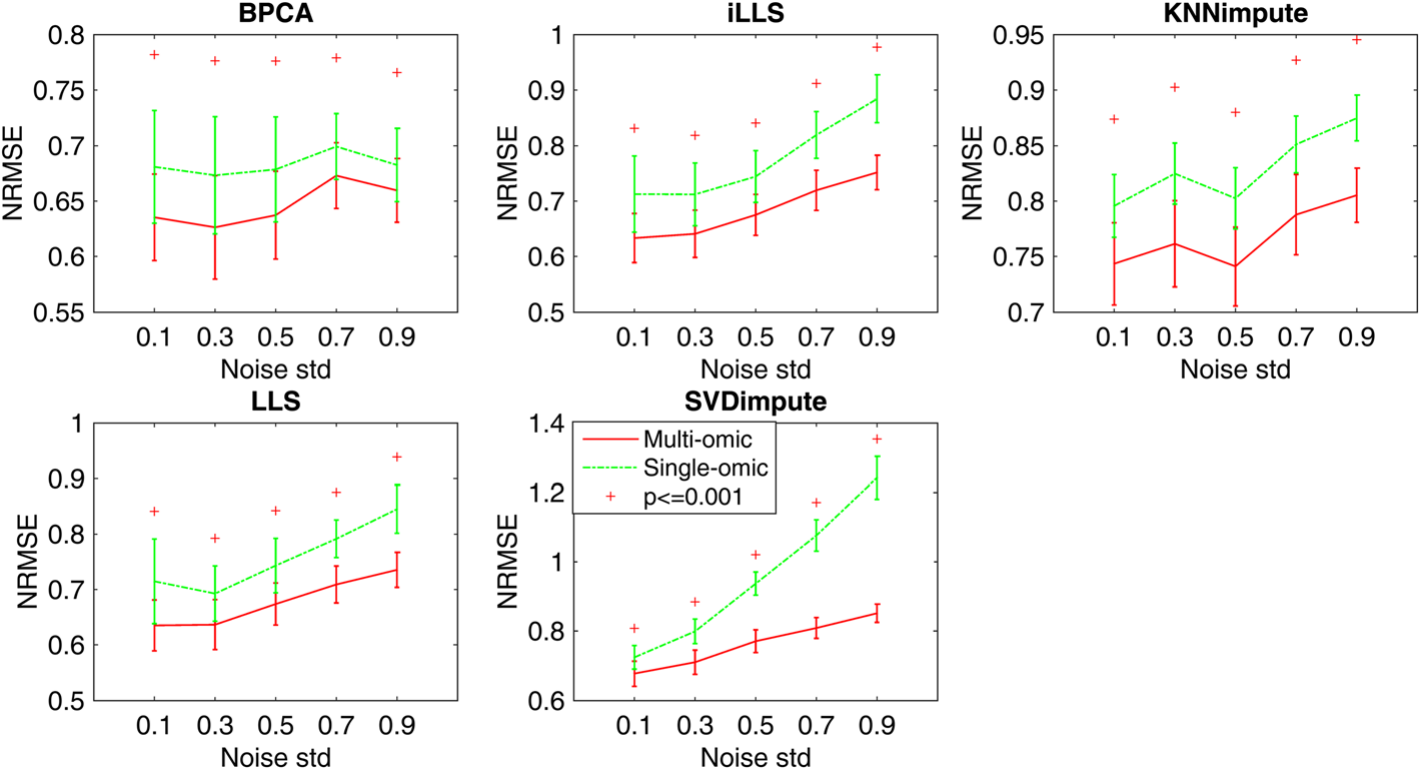
Average NRMSE by five imputation algorithms (BPCA, iLLS, KNNimpute, LLS and SVDimpute) on single omics data only v.s. multiple omics datasets with noise of different standard deviations (std) from 0.1 to 1

Figure 4 shows the comparison of single-omics and multi-omics imputation methods by varying noise level from 0.1 to 1.0. For all five algorithms, significant increase of NRMSE can be seen with the increasing level of noise. Our multi-omics method consistently outperforms single-omics method among all algorithms, showing the importance of using complementary information (mRNA and DNA methylation) to suppress noise in miRNA. In addition, with the increase of noise level (e.g., std > 0.5), the performance gain of multi-omics method over single omics method also increases, indicating its better robustness to noise.

### Comparisons on multiple missing omics data

To further evaluate the imputation methods when there are missing values in multiple omics data, we simulated mRNA and miRNA datasets with different missing rates of 1, 5 and 10 % respectively. Five single-omics methods were used to impute each missing data separately and then compared with our iterative multi-omics imputation method, which can impute both missing matrices simultaneously.

**Fig. 5:**
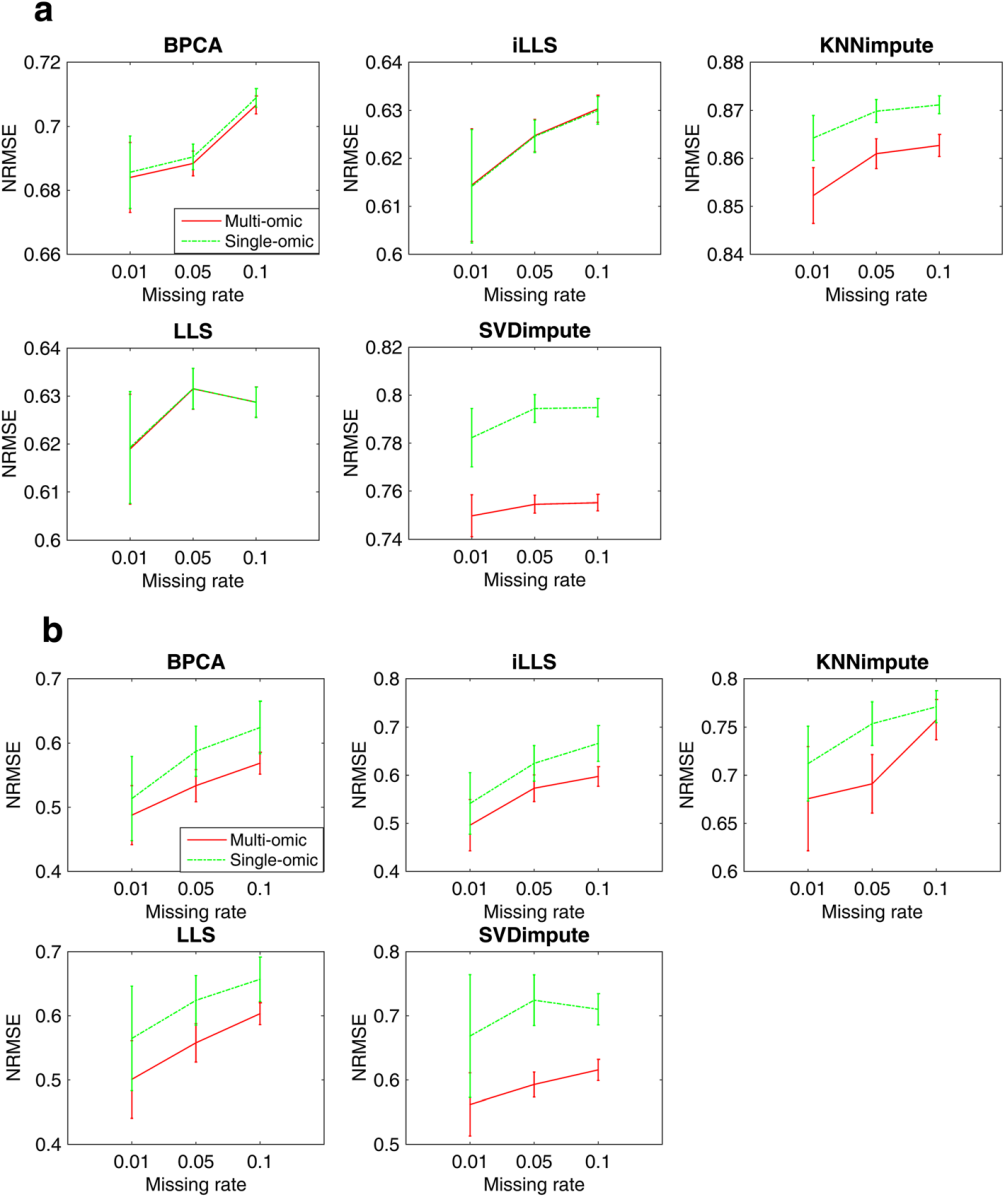
The NRMSE of iterative multi-omics imputation and five single-omics imputation methods on both **a** mRNA and **b** miRNA missing matrices

Figure 5 shows the comparison of NRMSE between our iterative multi-omics imputation method and other single-omics methods on imputing both mRNA (Fig. 5a) and miRNA (Fig. 5b) missing matrices. Similar to the results in Fig. 4, our multi-omics method can significantly decrease the NRMSE of miRNA matrix imputation than all five single-omics methods. This improvement is not consistently found in mRNA imputation, as shown in Fig. 5(a). Local imputation methods such as LLS and iLLS show no change of NRMSE between our method and single-omics method; however, they deliver much lower NRMSE value than the other three methods. This may be because local mRNA expressions were highly correlated and local features can provide enough information for imputation. Moreover, although correlation exists between miRNA and mRNA, because of the small number of miRNA probes, they are not expected to provide much extra information for mRNA imputation.

### Comparisons for network analysis

Besides the comparison of imputation accuracy between different methods, we further evaluated the influence of different imputation methods on downstream analysis, e.g., reconstruction of regulatory network between mRNA and miRNA, which is significant for exploring the interactions between different omics. There are a variety of methods proposed for reconstructing genetic regulatory networks. We applied a web tool, miRNA and genes integrated analysis (MAGIA) [44], to reconstruct fundamental post-transcriptional regulatory networks between miRNAs and mRNAs for Glioma cancer. The Pearson correlation was used as the measure of interactions between each pair of miRNA and mRNA expressions on the matched design. Then the combination of two target prediction algorithms (PicTar and PITA) was applied to predict the target of miRNA and thus the mRNA-miRNA regulatory network was built based on significant test on each interaction.

We evaluated network reconstruction as a binary classification task (prediction of absence or presence of mRNA-miRNA interaction). The original network was constructed by using completed matrices. Then for each network built on the imputed matrices, a receiver operating characteristics (ROC) curve was derived by varying correlation threshold. The area under curve (AUC) was calculated to evaluate the influence of different imputation methods on regulatory network. Higher AUC indicates better capability of preserving significant interactions while lower AUC means worse effect of imputation methods on network structure reconstruction.

**Fig. 6:**
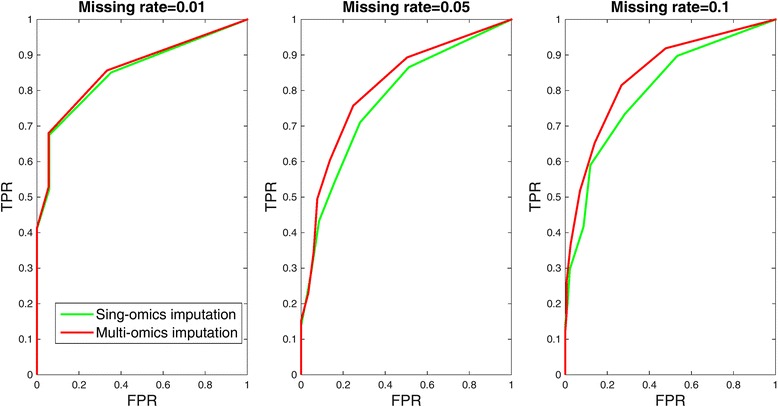
The ROC plots of identifying mRNA-miRNA interaction based on data imputed by iterative multi-omics imputation method and single-omics imputation (KNNimpute) method respectively. Missing rate changes from 0.01 to 0.1

Missing matrices of mRNA and miRNA were imputed and regulatory network was reconstructed by MAGIA using the correlation based algorithm. The original network structure was built based on complete matrices and their interactions (*p* < 1 × 10^−4^) were selected to be significant. There are 655 mRNA-miRNA interactions selected corresponding to the correlation value less than −0.55. For each pair of imputed mRNA and miRNA matrices by different methods, their interactions were reconstructed and compared with significant interaction set to obtain false positive rate (FPR) and true positive rate (TPR). Figure 6 shows the ROC plots, which compared the performance of iterative multi-omics imputation and single-omics imputation (i.e., KNNimpute) algorithms on recovering true mRNA-miRNA interactions. The curves were plotted by choosing different correlation thresholds. When missing rate is small, e.g., 1 %, both imputation methods give similar network structures with relatively better performance of multi-omics method for the two higher missing rates. The difference between two ROCs increases as the missing rate increases, showing the advantage of our iterative method for preserving significant mRNA-miRNA interactions

**Fig. 7:**
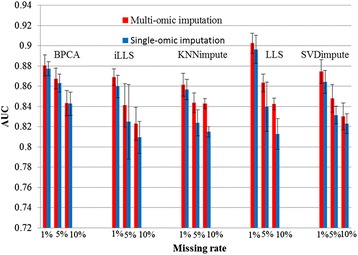
The AUC comparison between iterative multi-omics imputation and single-omics imputation methods by changing missing rate from 0.01 to 0.1

We further used AUC as a metric to compare their performances. The results shown in Fig. 7 were averaged on 15 replications. It can be seen that the missing value rate affects the network structure severely. High missing rate leads to reduced power of miRNA-mRNA identification (i.e., decrease of AUC) by applying single-omics imputation. However, our iterative imputation can compensate for this decrease by improving the imputation accuracy and utilizing correlations among omics data. This improvement was demonstrated consistently across all five algorithms, especially in local algorithms (e.g., KNNimpute, LLS, iLLS).

## Discussion

In this work, our multi-omics imputation method is able to combine the estimations from various basic models linearly to estimate missing values, e.g., self-imputation and cross-imputation. Multi-omics imputation method aims to employ information from different sources (e.g., diverse omics data). Each source is expected to contribute partially to the estimation. Similar to the contribution measure in [23], source contribution can be reflected in the coefficient weight in the final linear combination model as shown in (4). We evaluated the coefficient value of each source in the simulation of varying missing rate (data not shown). The weights of sources in most methods (except KNNimpute) are stable with the change of missing rate. miRNA shows higher weights than the other omics, indicating that self-information is still most important for imputation in this simulation. The other omics data also contain useful information and contribute to the improvement of imputation with appropriate weights. In addition to building basic model by different omics data, there are some other basic models such as multiple single-omics imputation algorithms that can also be incorporated as basic models and combined with cross-imputation estimation. Similar to the results discussed in [23], other types of single-omics imputation algorithms may also provide complementary information to improve imputation accuracy.

The cross-imputation part of the proposed multi-omics imputation method is based on regression model which requires the subjects cross omics data to be matched, while this case may not always hold in real data analysis. For the case that some subjects are shared across multiple omics data while some are omics data specific, we suggest the following imputation strategy: if those missing points are located on the subjects having only one type of omics data, only self-imputation part can be used in the proposed method, which degrades to single-omics imputation; for other missing points happened on subjects assayed with more than one types of omics data, both self-imputation and cross-imputation can be applied and combined in our method.

Since most of single-omics imputation methods were evaluated by simulations based on MCAR missing mechanism, we applied the same simulation strategy in this study for evaluation of multi-omics imputation performance. However, this assumption may not always hold in reality. Two other missing mechanisms (MAR and MNAR) may also be possible in expression data, as mentioned in Introduction. We further evaluated the performance of imputation methods with MAR missing mechanism by assuming data missing in some specific genes or proteins (e.g., existing on neighboring genes or probes). This missing may be due to the contamination of slides in the experiment, which can be documented for users. Additional file 1: Figure S1 shows the results of comparisons. There is no significant difference between two simulation schemes when missing rate is low (<0.2), indicating the insensitivity of the imputation methods to the way of how missing values are generated. When missing rate is high (i.e., 0.2), NRMSE in MAR imputation is much higher than that with MCAR missing, because of more information loss of these neighboring genes in MAR simulation (they all contain missing values). More importantly, the multi-omic method still shows significant improvement over single-omics methods since extra information can be provided by other omics data. For more complicated MNAR missing mechanisms, the loss of valuable information causing the missingness may make the most of imputation methods to be suboptimal and the estimation of missing patterns to be infeasible [1, 11]. Although this case is not discussed in this study, it is still expected that imputation method incorporating informative features from different types of omics data will outperform single-omics imputation methods, given the advantage of integrating more information from multiple omics data.

## Conclusions

We proposed a novel multi-omics imputation framework, which can take advantage of information from multiple omics data for improving the imputation accuracy. With the production of vast multi-omics data, there is increasing knowledge about complex biological relationship among multiple levels of omics (e.g., co-expression or co-regulation among gene expression, miRNA expression and transcriptional factors). We proposed multi-omics imputation method to exploit the underlying cross-omics relationship for missing value imputation. Experimental results confirmed the advantage of our multi-omics based method over five single-omics imputation methods (KNNimpute, BPCA, SVDimpute, LLS and iLLS) consistently in all three different scenarios in terms of lower value of NRMSE. To handle multiple omics data with missing values, we extended the imputation method, so it can utilize the relationship among multi-omics data iteratively to impute multiple missing omics data simultaneously. Compared with conventional single-omics methods for imputing each omics data separately, our iterative method is able to improve the imputation accuracy significantly in each missing omics data, especially for lower dimensional omics datasets, e.g., miRNA. In addition, the evaluation of mRNA-miRNA regulatory network demonstrated that our iterative method outperforms all five single-omics methods in uncovering the relationship across omics data, which is therefore significant for the study of biological regulatory mechanisms.

## Abbreviations

AUC, the area under curve; BPCA, Bayesian principle component analysis; FPR, false positive rate; GBT, Gradient Boosted Trees; GO, Gene Ontology; iLLS, iterative local least square; iMISS, integrative missing value estimation method; KNNimpute, k nearest neighboring impute; LLS, local least square; MAGIA, miRNA and genes integrated analysis; MAR, missing at random; MCAR, missing completely at random; MNAR, missing not at random; NRMSE, normalized root mean squared error; PPI, protein-protein interactions; ROC, the receiver operating characteristics curve; SVDimpute, singular value decomposition impute; TCGA, the cancer genomic atlas; TPR, true positive rate

## Additional file

Additional file 1: The document contains the brief introduction of various single-omics imputation methods, the algorithm of proposed multi-omics imputation method and the comparison of different imputation methods on simulation using MAR mechanism. (DOCX 122 kb)
