# Supplementary material for: An integrative imputation method based on multi-omics datasets
Source: BMC Bioinformatics. 2016 Jun 21;17:247. doi: 10.1186/s12859-016-1122-6 (PMC4915152; doi:10.1186/s12859-016-1122-6)
Supplement: Additional file 1: — The document contains the brief introduction of various single-omics imputation methods, the algorithm of proposed multi-omics imputation method and the comparison of different imputation methods on simulation using MAR mechanism. (DOCX 122 kb) [file 12859_2016_1122_MOESM1_ESM.docx]

Supplementary Information for

An integrative imputation method based on multi-omics datasets

Dongdong Lin^1,2†^, Jigang Zhang^2,3†^, Jingyao Li^1,2^,Chao Xu^2,3^, Hong-Wen Deng^2,3^, Yu-Ping Wang^1,2,3*^

^1^Biomedical Engineering Department, Tulane University, New Orleans, LA, 70118, USA

^2^Center of Genomics and Bioinformatics, Tulane University, New Orleans, LA, 70112, USA

^3^Department of Biostatistics and Bioinformatics, Tulane University, New Orleans, LA, 70112, USA

†:The authors wish it to be known that, in their opinions, the first two authors should be regarded as joint First Authors

*: To whom correspondence should be addressed.

Email addresses:

D.D. Lin: dlin5@tulane.edu

J.G. Zhang: jzhang9@tulane.edu

J.Y. Li: jli12@tulane.edu

C.X: cxu2@tulane.edu

H.W. Deng: hdeng2@tulane.edu

Y.P. Wang: [wyp@tulane.edu](mailto:wyp@tulane.edu)

**A:** **Brief description of each single omic imputation method**

As mentioned in ‘Methods’ section, we denote gene expression matrix as $G\in R^{p_{1}\times n}$ containing a target missing gene $g_{t}\in R^{1\times n}$, where $p_{1}$ is the number of total genes and n is the number of subjects. Without the loss of generality, we assume that missing values exist in the first s subjects of the target gene, denoted by $g_{t}=[g_{t}^{miss},g_{t}^{c}]$, where $g_{t}^{miss}\in R^{1\times s}$ is the missing vector in the target gene and $g_{t}^{c}\in R^{1\times(n-s)}$ is the complete vector without missing values. $Gk=[{Gk}^{miss},{Gk}^{c}]{\in R}^{k\times n}$ denotes the k nearest neighboring genes or eigengenes of target gene, where

$\left[ \begin{aligned} g_{t} \\ {Gk}_{1} \\ {Gk}_{2} \\ \vdots\\ {Gk}_{k} \end{aligned} \right]=\left[ \begin{aligned} g_{t}^{miss},g_{t}^{c} \\ {Gk}^{miss},{Gk}^{c} \end{aligned} \right]$  (A-1)

We briefly introduce each simple-omics imputation method to impute the missing target gene.

1. KNNImpute method

As proposed by Troyanskayan et.al, [[1](#_ENREF_1)] KNNImpute is to select the closest K genes of target gene to predict missing values in target gene. A similarity measure is necessary to find the closest K genes. The distance between the i-th gene and the target gene t is defined by the Euclidean distance as suggested, denoted by $d_{i,t}$

$d_{t,i}=\sum_{j\in C} \left( g_{t}^{j}-g_{i}^{j} \right)^{2}$ (A-2)

where $C$ is the set of indices for which there are no missing values in $g_{t}$. The smaller of $d_{i,t}$, the closer of the i-th gene to the target gene. Then, K closest genes are selected ($Gk$) to estimate the missing values in the target gene $g_{t}^{miss}$ by averaging the weighted values of the elements at the missing indices as follows:

$\tilde{g}_{t}^{miss}=\frac{\sum_{i=1}^{k} {Gk}_{i}^{miss}/d_{t,i}}{\sum_{i=1}^{k} 1/d_{t,i}}$ (A-3)

1. Local least square method

There are two steps for LLS imputation [[2](#_ENREF_2)]. The first step is to select k nearest neighboring genes using the distance measure defined similar to KNNimpute method. The row average method was usually applied to impute missing values initially and then those coherent genes were selected by their distance to the target gene. The missing values at the target gene are estimated by these coherent genes using regression based method as follows

$g_{t}^{c}={Gk}^{c}\times\beta$ (A-4)

where non-missing values in both the target gene and its k nearest neighboring genes were used to estimate the coefficients. The coefficient vector $\beta$ can be solve by ordinary least square as

$\beta=\left( {Gk}^{c} \right)^{\dagger}g_{t}^{c}$ (A-5)

where $\left( {Gk}^{c} \right)^{\dagger}$ is the pseudoinverse of matrix ${Gk}^{c}$. The missing values $g_{t}^{miss}$ can be estimated by

$\tilde{g}_{t}^{miss}={Gk}^{miss}\times\beta$ (A-6)

1. Iterated local least square method

iLLSimpute [[3](#_ENREF_3)] is built on LLS method but run imputation multiple times on the same target gene to approach a convergent estimation. At each iteration, LLSimpute was applied to impute missing values of target genes. Then based on imputed matrix, for each target gene, iLLS re-select coherent genes and re-run LLSimpute method. This procedure is implemented iteratively until a pre-specified number of iterations is achieved or there are no differences of imputed values between two iterations.

1. Singular Value Decomposition (SVD) imputation

Instead of selecting coherent genes, SVDimpute [[1](#_ENREF_1)] applies singular value decomposition to gene expression matrix to obtain a set of mutually orthogonal expression patterns. The decomposed expression patterns are then taken as eigngenes to impute missing values in target genes. Usually missing values in gene expression matrix need to be imputed (e.g.,by row average method) for SVD decomposition. $G^{p\times n}=U_{p\times p}D_{p\times n}V_{n\times n}^{T}$, where $V_{n\times n}^{T}$ is eigengene matrix. K nearest neighboring eigngenes are selected by same procedure as above and fit in a regression model with target gene expression as A-4~6 to estimate missing values.

1. Bayesian Principle Component Analysis (BPCA)

BPCA [[4](#_ENREF_4)] represents the target gene vector as a linear combination of K principle axis vectors $v_{l}$ as

$g_{t}=\sum_{l=1}^{K} u_{l}v_{l}+\varepsilon$ (A-7)

where $u_{l},l=1,2,\ldots,K$ is factor scores and $\varepsilon$ is residual error. A probalistic model is applied by assuming that $u$ follows normal distribution $u\sim N\left( \mu,I_{K} \right)$ and $\varepsilon\sim N\left( 0,{\frac{1}{\tau}I}_{p1} \right)$. The model parameters $\theta=\left( v,\mu,\tau\right)$ is estimated by Bayesian estimation method. An automatic relevance determination (ARD) prior is designed for the coefficient vector $v_{l}$. An EM-like algorithm is then used to estimate the posterior distributions of $\theta$ and the missing values simultaneously as

$q\left( g_{t}^{miss} \right)=\int d\left( \theta\right)q\left( \theta\right)p(g_{t}^{miss}|g_{t}^{c},\theta)$ (A-8)

$q\left( \cdot\right)$ indicates the posterior distribution. Finally, the missing value can be estimated to the expectation with respect to the estimated posterior distribution by

$\tilde{g}_{t}^{miss}=\int q\left( g_{t}^{miss} \right)g_{t}^{miss}d{(g}_{t}^{miss})$ (A-9)

**B. Optimization algorithm and implementation**

Equation 4 is can be reformulated by matrix as

$$\min\left\| \boldsymbol{y}-\boldsymbol{A}\boldsymbol{\beta} \right\|_{2}$$

$$s.t. \boldsymbol{c}^{T}\boldsymbol{\beta=1;}\boldsymbol{\beta}_{\boldsymbol{i}}\geq0$$

where $\boldsymbol{y=}{\boldsymbol{[}\boldsymbol{G}_{1}^{1}\boldsymbol{,}\boldsymbol{G}_{1}^{2}\boldsymbol{,\ldots,}\boldsymbol{G}_{1}^{J}\boldsymbol{]}}^{\boldsymbol{T}}$**,** $\boldsymbol{A=}\left[ \boldsymbol{A}_{\boldsymbol{1,1,}}\boldsymbol{A}_{\boldsymbol{1,2,}}\boldsymbol{\ldots,}\boldsymbol{A}_{\boldsymbol{i,b,}}\boldsymbol{,\ldots,}\boldsymbol{A}_{\boldsymbol{3,B}} \right]\boldsymbol{,i=1,2,3;b=1,2,\ldots,B}$**;** $\boldsymbol{A}_{\boldsymbol{i,b,}}\boldsymbol{=}\left[ \boldsymbol{G}_{\boldsymbol{1\leftarrow i,b}}^{1}\boldsymbol{,}\boldsymbol{G}_{\boldsymbol{1\leftarrow i,b}}^{2}\boldsymbol{,\ldots,}\boldsymbol{G}_{\boldsymbol{1\leftarrow i,b}}^{J} \right]^{\boldsymbol{T}}$**;** $\boldsymbol{\beta=}\left[ \boldsymbol{\beta}_{\boldsymbol{1}}\boldsymbol{,}\boldsymbol{\beta}_{\boldsymbol{2}}\boldsymbol{,\ldots,}\boldsymbol{\beta}_{\boldsymbol{3}\boldsymbol{B}} \right]^{\boldsymbol{T}}$**;**$\boldsymbol{c=}\left[ 1,1,\boldsymbol{\ldots},1 \right]^{\boldsymbol{T}}$**.** The optimization is a constrained least square minimization problem including quadratic object function, linearly equality constraint and a set of inequality constrains (non-negative constraints). This convex problem can be solved by numerical algorithms for quadratic programming.

Active set method is popularly used, especially for non-negative least square minimization [[5](#_ENREF_5)]. It begins with setting a set of constraints as active set, and then iteratively adds or removes set of constraints to minimize the object function. The method is applied to our problem and implemented by MATLAB function ‘lsqlin’.

**C. Simulation with missing at random (MAR) mechanism**

In this simulation, we aimed to simulate missing values based on MAR mechanism. We changed the simulation scheme by simulating missing values only on some nearby genes (e.g., 10 neighboring genes) with varying missing rate from 1% to 20%. Both single-omic and multi-omic imputation methods were compared. The result is shown in Figure S1. It can be seen that NRMSE increases along with increasing missing rate, especially a jump ascent when the missing rate is 0.2. However, our proposed method still significantly outperforms the single-omics imputation methods in terms of lower missing errors, which is consistent with our results in the simulation based on missing completely at random (MCAR) mechanism.


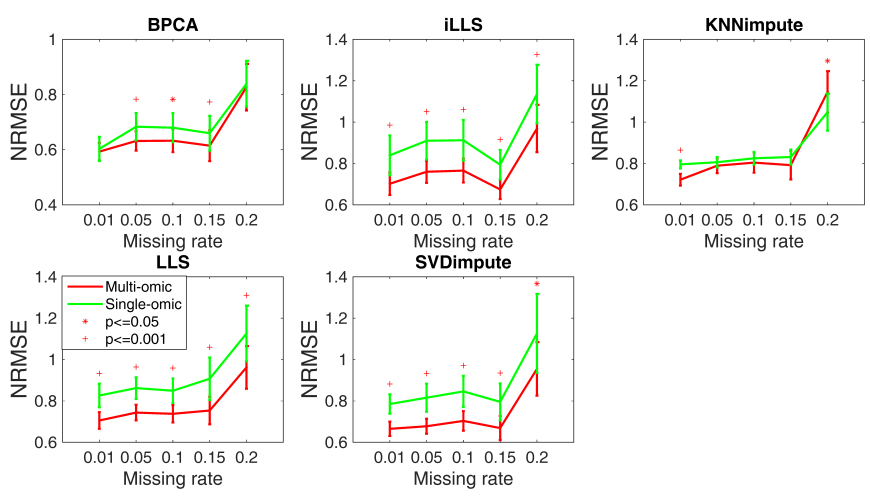


Figure S1. Comparison of imputation methods on missing data generated by missing at random (MAR) mechanism with varying missing rate from 0.01 to 0.2.

1. Troyanskaya O, Cantor M, Sherlock G, Brown P, Hastie T, Tibshirani R, Botstein D, Altman RB: **Missing value estimation methods for DNA microarrays**. *Bioinformatics* 2001, **17**(6):520-525.

2. Kim H, Golub GH, Park H: **Missing value estimation for DNA microarray gene expression data: local least squares imputation**. *Bioinformatics* 2005, **21**(2):187-198.

3. Cai Z, Heydari M, Lin G: **Iterated local least squares microarray missing value imputation**. *Journal of bioinformatics and computational biology* 2006, **4**(05):935-957.

4. Oba S, Sato M-a, Takemasa I, Monden M, Matsubara K-i, Ishii S: **A Bayesian missing value estimation method for gene expression profile data**. *Bioinformatics* 2003, **19**(16):2088-2096.

5. Lawson CL, Hanson RJ: **Solving least squares problems**, vol. 161: SIAM; 1974.
